# Implementation of a zero fluoroscopic workflow using a simplified intracardiac echocardiography guided method for catheter ablation of atrial fibrillation, including repeat procedures

**DOI:** 10.1186/s12872-021-02219-8

**Published:** 2021-08-26

**Authors:** Tamas Tahin, Adam Riba, Barnabas Nemeth, Ferenc Arvai, Geza Lupkovics, Gabor Szeplaki, Laszlo Geller

**Affiliations:** 1 Department of Cardiology, Zala County St. Rafael Hospital, Zrinyi str. 1, Zalaegerszeg, 8900 Hungary; 2 Heart and Vascular Centre, Semmelweis University, Budapest, Hungary; 3 Heart and Vascular Centre, Mater Private Hospital, 72 Eccles Street, Dublin 7, Ireland; 4 Royal College of Surgeons in Ireland, Dublin, Ireland

**Keywords:** Atrial fibrillation, Pulmonary vein isolation, Intracardiac echocardiography, Fluoroscopy-free

## Abstract

**Objective:**

Pulmonary vein isolation (PVI) is the cornerstone of the interventional treatment of atrial fibrillation (AF). Traditionally, during these procedures the catheters are guided by fluoroscopy, which poses a risk to the patient and staff by ionizing radiation. Our aim was to describe our experience in the implementation of an intracardiac echocardiography (ICE) guided zero fluoroscopic (ZF) ablation approach to our routine clinical practice.

**Methods:**

We developed a simplified ICE guided technique to perform ablation procedures for AF, with the aid of a 3D electroanatomical mapping system. The workflow was implemented in two phases: (1) the Introductory phase, where the first 16 ZF PVIs were compared with 16 cases performed with fluoroscopy and (2) the Extension phase, where 71 consecutive patients (including repeat procedures) with ZF approach were included. Standard PVI (and redoPVI) procedures were performed, data on feasibility of the ZF approach, complications, acute and 1-year success rates were collected.

**Results:**

In the Introductory phase, 94% of the procedures could be performed with complete ZF with a median procedure time of 77.5 (73.5–83) minutes. In one case fluoroscopy was used to guide the ICE catheter to the atrium. There was no difference in the complication, acute and 1-year success rates, compared with fluoroscopy guided procedures. In the Extension phase, 97% of the procedures could be completed with complete ZF. In one case fluoroscopy was used to guide the transseptal puncture and in another to position the ICE catheter. Acute success of PVI was achieved in all cases, 64.4% patients were arrhythmia free at 1-year. Acute major complications were observed in 4 cases, all of these occurred in the redo PVI group and consisted of 2 tamponades, 1 transient ischemic attack and 1 pseudoaneurysm at the puncture site. The procedures were carried out by all members of the electrophysiology unit in the Extension phase, including less experienced operators and electrophysiology fellows (3 physicians) under the supervision of the senior electrophysiologist. Consequently, procedure times became longer [90 (75–105) vs 77.5 (73.5–85) min, *p* = 0.014].

**Conclusions:**

According to our results, a ZF workflow of AF ablations can be successfully implemented into the routine practice of an electrophysiology laboratory, without compromising safety and effectivity.

## Introduction

Atrial fibrillation (AF) is the most common arrhythmia and with the aging patient population, it has a growing impact on the healthcare systems. The most advanced treatment of atrial fibrillation is pulmonary vein isolation [1, 2]. The therapy has an average success rate of 70–80% [3, 4]. The increasing number of pulmonary vein isolation procedure lays a significant burden for healthcare professionals. One of the most important hazards related to the procedure is radiation exposure [5]. Other factors affecting the health of the operating personnel are musculoskeletal diseases [6]. Thus, the aim of reducing the ionizing radiation exposure to the patients and staff is a very important.

Conventionally, electrophysiological procedures rely on analyzing intracardiac signals (EGMs) and the catheters are guided by fluoroscopy. To reduce radiation exposure, the as low as reasonably achievable (ALARA) radiation safety principle should be followed. The concept is based on the minimization of radiation doses and limiting the release of radioactive materials into the environment by employing all “reasonable methods.” Due to the stochastic effects of radiation, there is no safe lower threshold, thus completely avoiding fluoroscopy is the only way to completely avoid radiation hazards. Moreover, the orthopedic problems related to the use of lead apparel cannot be omitted.

Efforts to reduce fluoroscopy include pre-intervention magnetic resonance imaging (MRI) or computer tomography (CT) imaging, transesophageal or intracardiac echocardiography (TEE or ICE) and the use of electroanatomical mapping systems (EAMs) [7–14]. However, none of the mentioned modalities lead to completely zero-fluoro procedures [15, 16].

We made continuous efforts to reduce the use of fluoroscopy during the ablation of AF in past years. With gradually reducing fluoroscopy use during the procedures, eventually got to milestone, where fluoroscopy was used only during the transseptal puncture (TSP). Therefore, we have aimed to introduce a new method for a complete zero fluoroscopic TSP, that can avoid the hazards related to ionizing radiation. In the present article, our aim was to demonstrate the safety and feasibility of this refined ICE guided transseptal technique in a consecutive patient cohort, who underwent AF ablation (including those with repeat procedures).

## Methods

### Study design and patient population

The study consisted of two main phases: (1) the Introductory phase, where the aim was to test the safety and feasibility of the zero-fluoroscopy (ZF) technique versus the conventionally used, fluoroscopy guided (FL) workflow and (2) the Extension phase, where the longer-term outcomes were tested using the ZF strategy in consecutive patients. A total of 103 patients were enrolled into the study between January 2017 and December 2018, who underwent catheter ablation by pulmonary vein isolation (PVI) for symptomatic, non-valvular atrial fibrillation at our Centre. Thirty-two patients were enrolled to the Introductory phase, who have undergone either a ZF or a FL PVI. The fluoroscopic strategy was alternating case-by-case; a ZF case was followed by a FL case until the total case numbers were met. All cases had been performed by one independent senior operator, with > 20 years of work experience. After the first phase, the next 71 consecutive patients with atrial fibrillation ablation underwent a ZF strategy in the Extension phase. Among them we included 29 patients with prior PVI who underwent a redo-PVI procedure. The procedures were carried out by all members of the electrophysiology unit, including less experienced operators and electrophysiology fellows (3 physicians) under the supervision of the senior electrophysiologist. The indication for the ablation procedures was in accordance with the current European Society of Cardiology guidelines [17]. Data analysis was done retrospectively, the study was in accordance with the Declarations of Helsinki and the protocol was approved by local ethics board.

### Catheter ablation procedure

All procedures were done in conscious sedation using iv. Fentanyl, midazolam and propofol for analgesia. The procedures were carried out on uninterrupted anticoagulation, with a target INR < 3 for patients on coumarine or warfarin, while the morning dose was held for patients on direct anticoagulants. Intracardiac electrocardiography (ICE) imaging was used to rule out the presence of thrombus in the left atrium (LA). For the standard FL guided procedures only, a decapolar reference catheter was inserted into the coronary sinus (Dynamic Deca, Boston Scientific) via the femoral vein. A double transseptal puncture was done with non-steerable sheaths with continuous pressure monitoring using the Brockenbrough’s technique [18]. For the FL group the puncture was guided by fluoroscopy and ICE; the ZF method is presented below in details. After double transseptal access patients were heparinized with a target ACT of 250–350 s. LA was not reconstructed by any imaging modalities (computer tomography etc.) prior the index procedure. The LA was mapped by a circular 20-pole Lasso catheter (Biosense Webster Inc.) using the fast anatomical mapping module of the CARTO3™ electroanatomical mapping system (Biosense Webster Inc.). Then the pulmonary veins were isolated in pairs using bilateral wide antral circumferential ablation with an irrigated contact force sensing ablation catheter (ThermoCool SmartTouch™ 8Fr, Biosense Webster). For the redo-PVI procedures, the gaps were targeted only with RF applications until re-isolation was achieved. No additional ablation technique apart from PVI was done in any of the cases included in the present study. Acute procedural success was defined as PVI validated by entrance and exit block after a 20-min waiting period.

### Simplified zero-fluoroscopic transseptal puncture

**Fig. 1:**
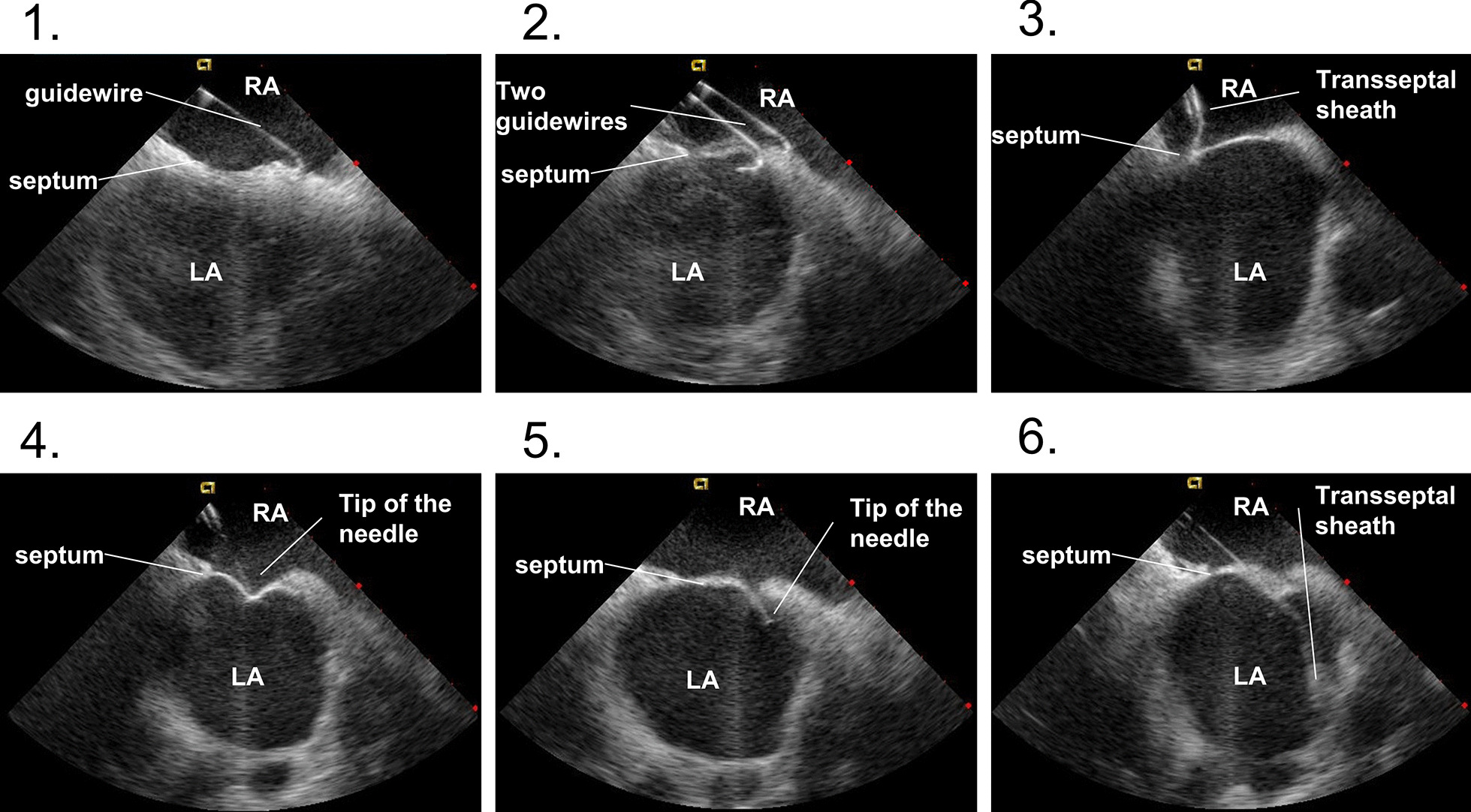
Representative ICE images during the most important steps during the zero-fluoroscopic transseptal puncture. See text for detailed description of the technique. Abbreviations used: LA—left atrium, RA—right atrium

A 10F ICE catheter (ACCUSON AccuNav, Biosense Webster) is advanced to the right atrium (RA) without the use of fluoroscopy via the left femoral vein—the ultrasound image can help to navigate the catheter up to the RA in real time. Next, two long J wires are placed into the RA via the right femoral vein and their position is confirmed by the ICE image (Fig. 1, step 1). Then the tip of the ultrasound catheter is deflected posteriorly in order to allow visualization of the thinnest, optimal target part of the interatrial septum. The distal end of the long J wire is placed at the fossa ovalis (Fig. 1, step 2) and the first transseptal sheath is advanced over the wire at 5 o’clock direction face and placed at the interatrial septum (Fig. 1, step 3). Normally, tenting of the septum can be seen promptly and the transseptal sheath is fixed at the desired area of the septum with the help of the dilator. Next, the Brockenbrough needle (BRK, Abbott) is inserted into the transseptal sheath (Fig. 1, step 4). Then the transseptal puncture is performed with continuous pressure and ICE monitoring (Fig. 1, step 5)—usually this can be achieved with minimal resistance while crossing the septum. Finally, the sheath as advanced through the septum with visual guidance and the dilator with the needle is removed (Fig. 1, step 6). The second transseptal puncture is done with the exact same methodology.

### Data collection and follow-up protocol

Patient demographics, medical history and atrial fibrillation specific data was recorded at baseline prior to the ablation procedure. The total skin-to-skin procedure time was noted, while in the Introductory phase its components also (ICE catheter placement, ICE examination, advancement of wires and sheaths, transseptal puncture, mapping and validation) were recorded. In case where a ZF procedure had to be converted to fluoroscopy, the reason was noted. Fluoroscopy times for such cases and for all FL procedures were recorded. Acute procedural success was defined as bilateral PVI confirmed by entrance and exit block after the 20 min validation period. All procedural and early-postprocedural complications were recorded before discharge, the definitions for the complications were in line with the most recent consensus documents [19]. Patients were followed-up for 1 year, with outpatient clinic visits at 3, 6, 9 and 12 months. At these visits relevant new medical history and symptoms were notes, a standard 12-lead ECG recording was done and patients had a 24 h continuous Holter ECG monitoring. Recurrence of atrial fibrillation was defined as a > 30 s episode of atrial fibrillation or atrial tachycardia (excluding typical atrial flutter) which was documented after the 3 months post-ablation blanking period.

### Statistical analysis

Statistical analysis was performed with the SPSS Statistics software package (IBM, NY, USA). Continuous dependent variables were tested for normality with Shapiro-Wilks test. The homogeneity of the groups was tested by F-test (Levene’s test). Continuous variables with normal distributions were compared with the Student’s *t*-test, while those with non-Gaussian distributions were tested with the Mann–Whitney *U* test. All tests were performed two-tailed and a *p* < 0.05 was considered as significant. Variables in the text are presented as medians and interquartile ranges, means + SEM or absolute numbers (%).

## Results

### Results of the introductory phase

There were no significant differences in demographics, comorbidities or type of AF between the ZF and FL patient groups (Table 1).

**Table 1:** Patient demographics in the introductory phase

	Introductory phase		*p* Value
	ZF (n = 16)	FL (n = 16)	
Age, years	57.9 ± 2.8	59.4 ± 1.8	0.647
Female gender	5 (31.3%)	2 (12.5%)	
BMI, kg/m^2^	28.6 ± 1.0	28.5 ± 1.3	0.969
Hypertension	11 (68.8%)	9 (56.3%)	0.716
Diabetes mellitus	1 (6.3%)	4 (25%)	0.333
Coronary artery disease	1 (6.3%)	5 (31.3%)	0.172
Prior stroke/TIA	0	0	
Persistent atrial fibrillation	3 (18.8.25%)	4 (25.0%)	1.000
*EHRA classification*			
1	0	0	
2	13 (81.3%)	11 (68.8%)	0.343
3	3 (18.3%)	5 (31.3%)	0.343
4	0	0	
Sinus rhythm at baseline	13 (81.3)	9 (56.3%)	0.252
LA diameter (mm)	41.2 ± 2.2	45.13 ± 2.2	0.211

*ZF* zero-fluoroscopic group, *FL* fluoroscopic group, *TIA* transient ischemic attack. Values are mean ± SEM and absolute numbers

The complete ZF has been successfully done in 94% of the cases. In one case we had to convert to the use of fluoroscopy, due to the difficulties in advancing the ICE catheter through the femoral vein to the right atrium. Acute procedural success rate (PVI) was 100% in both subgroups. Fluoroscopy time was significantly shorter in the ZF group, while there was no significant difference in the total procedure times between the two groups (Table 2). No major complications occurred either in the ZF or the FL group during this phase, minor hematoma was observed in one case in the ZF group.

**Table 2:** Detailed procedure times of each step in the introductory phase

	ZF (n = 16)	FL (n = 16)	*p* Value
Total fluoroscopy time (s)	0 (0–0)	370 (269–442.5)*	< 0.001
Procedure time (min)	77 (73.5–85.5)	77.5 (73–84.5)	0.724
Introducing the ICE catheter (min)	4 (4–5)	4.5 (3–6)	0.616
ICE examination (min)	6 (4.25–7.75)	5 (4–5)	0.119
Advancing wires and sheaths (min)	5.5 (4–7.5)	5 (4–6)	0.780
Transseptal puncture (min)	7 (5.25–9)	8 (5–8)	0.897
Mapping (min)	7 (5–8)	8 (6.25–8.75)	0.305
Ablation (min)	28 (25.25–31.25)	29 (21.25–33.75)	0.92
Validation (min)	20	20	

*ZF* zero-fluoroscopic group, *FL* fluoroscopic group. Values are medians (interquartile ranges), *p < 0.05

### Outcomes of the Extension Phase

In the Extension phase, 71 consecutive patients with atrial fibrillation ablation were enrolled, including 42 patients who had their first PVI procedure and 29 patients, who underwent redo PVI.

Procedure times were significantly longer in this second phase [90 (75–105) vs. 77.5 (73–85) min, *p* = 0.014]. There were no differences in the demographic parameters between the de novo PVI (First PVI) and the redo (Redo PVI) ablation groups, but left atrial size was significantly larger in the redo PVI group.

Complete zero fluoroscopy was achieved in 97% of the cases in the Extension phase. Two cases had to be converted to the use of fluoroscopy; on one occasion fluoroscopy was required to advance the ICE catheter to the right atrium due to the tortuosity of the femoro-iliac venous system, but the transseptal puncture was done without fluoroscopy. In one other case the septum could not been properly visualized, and fluoroscopy was required to perform the transseptal puncture. Acute procedural success (PVI) was 100%.

Acute major complications were observed in 4 cases (5.6%). All of these occurred in the redo PVI group and consisted of 2 tamponades, 1 TIA and 1 pseudoaneurysm at the puncture site. One tamponade occurred during the isolation of the right sided pulmonary veins, ablating at the superior area. ICE is particularly helpful in the early detection of pericardial effusion. We noticed a small effusion during the procedure, but we evaluated the risk of tamponade and decided to proceed with the procedure. Due to the growing pericardial effusion and clinical signs of tamponade pericardiocentesis had to be performed and the pericardium was drained. The pericardial cannula was removed the morning after the procedure and the patient was discharged the following day. One tamponade occurred after withdrawing the transeptal sheaths from the left atrium following a complete PVI. This patient was treated with pericardiocentesis and did not require surgery either. In one case TIA was noted after the recovery from the procedure, but the neurological symptoms have resolved by the following day without any residual symptoms. In one case pseudoaneurysm of the femoral artery was detected after the procedure, which successfully treated with thrombin injection. One patient developed minor hematoma of the groin, not requiring transfusion/surgery.

**Fig. 2:**
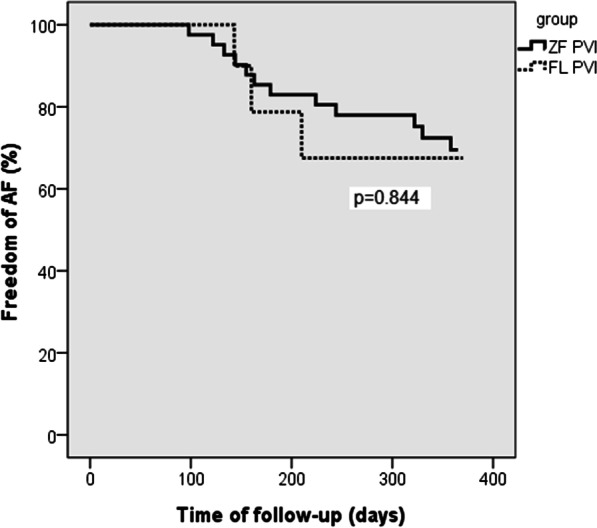
Freedom from AF during 1-year follow-up ZF PVI versus FL PVI

No procedure related deaths, stroke/TIA events, atrio-esophageal fistula, phrenic nerve palsy, pulmonary vein stenosis or other late complications of the catheter ablation were observed during the one year follow-up period. Complete data on AF recurrence, including serial Holter recordings was available in 90 cases (87%). There was no significant difference between the FL and ZF PVI (including both introductory and extension phase patients) subgroups success rates at 1-year (ZF PVI: 76.0% vs. FL PVI: 76.9%, *p* = 0.844, Fig. 2). The 1-year success rate in the ZF redo PVI group was 44.4%.

## Discussion

We have described a simplified ICE guided AF ablation technique, with the aim for zero-fluoroscopy throughout the procedures. The study consisted of an Introductory phase, with limited number of patients included and where a direct comparison was made to a control group of conventional fluoroscopy guided procedures. This was followed by the Extension phase, where the new workflow was adopted to by other staff members on a consecutive AF population undergoing catheter ablation, including repeat ablations. According to our results, complete zero-fluoroscopy could be achieved in 94% and 97% of the cases of the respective phases. No procedure related increase in major complications was observed. The novel technique did not significantly prolong procedure times nor it affected short and longer-term efficacy of the ablations. Thus feasibility, safety and efficacy were reliably demonstrated using this method.

The role of electroanatomic mapping system guided zero-fluoroscopy ablations has been increasing in the recent years, with many centers routinely performing this technique, mainly for the treatment of supraventricular tachycardias [20, 21]. Previous studies have shown, that these procedures can significantly reduce the amount of ionizing radiation, which can ultimately lead to a decreased risk of cancer incidence and mortality [20–22]. As these procedures can be performed without wearing radioprotective lead aprons, a reduction in orthopedic hazards can be anticipated as well. Though reported rates for complete zero-fluoroscopy in right atrial procedures can be as high as 100% (Walsh2018), in some cases conversion to the use of fluoroscopy is required. The overall complete zero-flurosocopy rate varies between 70–95% among different SVT cohort, however the significant reduction in radiation dose is observed in those cases as well, where zero-fluoroscopy was only attempted (ie. minimal fluoroscopic cases) [20–22]. The main limiting step for the complete zero-fluoroscopic procedures in left atrial arrhythmias is the safe performance of a transseptal puncture for access—which also applies for AF.

The overcome that limitation, we have optimized the ICE guided transseptal puncture, by directly advancing the wires and sheaths to the septum from the inferior vena cava, without the use of EAM. Previously reported techniques related to zero-fluoro transseptal puncture proposed the requirement of at least using EAM system to map the RA prior to the puncture wich is guided by TOE or ICE [23]. The use of TOE can be an optimal solution in patients under general anesthesia, however it would be challenging in our setting, where AF ablations are done in conscious sedation. Moreover, one previous study has revealed, that by using TOE procedure, the probability of esophageal hematoma formation can be higher [9]. The use of TOE versus ICE might seem to be cost effective at first glance considering the direct costs, but as it requires general anesthesia and an extra staff to operate the probe, the associated indirect costs might be underestimated. [12, 24]. The use of patent foramen ovale to access the left atrium can be an alternative as well, which avoids transseptal puncture, but it is only feasible in limited amount of cases [25]. Thus in our environment, the only feasible technique was ICE imaging. The previously reported “ICE only” transseptal puncture techniques consecutively used the EAM (usually an ablation catheter inserted into the transseptal sheath) to record RA anatomy [23]. This double imaging technique was used to facilitate ICE imaging in the transseptal puncture. Using our approach, the transseptal puncture was feasible in > 95% of the cases, with potentially less time spent on that part of the procedure. Moreover, introducing or exchanging instruments in the transseptal sheath can considerably increase the risk of thromboembolic complications during the intervention. No such complication was observed in our cohort.

Our journey to implement a completely new workflow in our EP laboratory started with the Introductory phase, where one highly experienced electrophysiologist performed all the procedures. The initial experience (including the learning curve) showed, that the new method is not inferior to the conventional one in terms of safety and acute efficacy. However, one commonly used argument against the zero-fluoroscopic approach might, that it requires highly skilled operators, with a lot of experience—hence it is difficult to integrate the approach into the daily practice. Thus, in our Extension phase we aimed to integrate the approach to the routine practice in our laboratory with success ever since. Although, when less experienced operators started to perform the procedures, a slightly increased procedure time was observed, but that did not affect the overall productivity of our laboratory.

Our study is the first, where the zero-fluoroscopic approach was described in consecutive patients scheduled for AF ablation, including repeat ablation procedures. Former reports were mainly focusing on first PVI procedures and apart from case reports, the feasibility of the fluoroscopy free approach in redo AF procedures has not been extensively described yet [26]. According to our first limited experience, repeat AF ablation procedures can also be successfully performed used this novel technique. However, it is important to note, that the only major complication (a case of a pericardial tamponade), which was possibly related to the zero fluoroscopic transseptal procedure occurred during a repeat procedure. It is well known in the clinical practice, that transseptal puncture can be complicated in a patient with prior left atrial procedure, possibly due to the scarring in the septum, which results in an increased resistance, when crossing the septum. Based on our limited experience, we suggest that in cases where the resistance is increased or the septum cannot be appropriately visualized by ICE, conversion to fluoroscopy is recommended. Literature is scarce on the procedural complication rates of repeat AF ablation procedures. Szegedi et al. [27] has described that a repeat procedure is an independent predictor of major complications, based on data obtained from a large cohort of AF ablations. This was reflected by our patient population as well. Thus, repeat procedures should be performed with extreme caution to avoid unnecessary complications.

## Limitations

The present manuscript reports on a single center, non-randomized observational study with limited number of patients included. Accordingly, due to the low number of complications, the results should be interpreted with caution.As the patient number was relatively low this limits the interpretations of conclusions on safety. Overall 1-year success rates for PVI might seem to be lower than those in some recently published studies, however some technologies (including ablation index guidance) were not available at the time at our laboratory. Despite fluoro dose is an important parameter, we did not collect it in this study and reported fluoroscopy times only. Fluoro times correlate much better with the technique used rather than the radiation dose which is altered by the patient body parameters. Further larger scaled studies are required to confirm our initial observations.

## Data Availability

Data available upon request from the corresponding author.

## Abbreviations

ALARA: As low as reasonably achievable
AF: Atrial fibrillation
CT: Computer tomography
EAM: Electroanatomical mapping
ECG: Electrocardiography
EGM: Electrocardiogram
FL: Fluoroscopy
ICE: Intracardiac echocardiography
MRI: Magnetic resonance imaging
PVI: Pulmonary vein isolation
TIA: Transient ischaemic attack
TEE: Transesophageal echocardiography
TSP: Transseptal puncture
ZF: Zero fluoroscopy
